# Targeting peripheral blood pro-inflammatory cytotoxic lymphocytes by inhibiting CD137 expression: novel potential treatment for COPD

**DOI:** 10.1186/1471-2466-14-85

**Published:** 2014-05-15

**Authors:** Greg Hodge, Mark Holmes, Hubertus Jersmann, Paul N Reynolds, Sandra Hodge

**Affiliations:** 1 Lung Research, Hanson Institute and Department of Thoracic Medicine, Royal Adelaide Hospital, Adelaide, South Australia; 2 Department of Medicine, University of Adelaide, Adelaide, South Australia

**Keywords:** COPD, Blocking CD137, CD28null T and NKT-like cells, NK cells, IFNγ and TNFα

## Abstract

**Background:**

We have shown that chronic obstructive pulmonary disease (COPD) is associated with increased production of pro-inflammatory cytokines and the cytotoxic mediator, granzyme B by peripheral blood steroid resistant CD28nullCD137 + CD8+ T cells and granzyme B by NKT-like and NK cells. We hypothesized that we could target these pro-inflammatory/cytotoxic lymphocytes by inhibiting co-stimulation through CD137.

**Methods:**

Isolated PBMC from patients with COPD and healthy controls were stimulated with phytohaemagglutinin (PHA) ± blocking anti-CD137 ± 10^-6^ M methylprednislone (MP) (±stimulatory anti-CD137 ± control antibodies). Pro-inflammatory **c**ytokine profiles and expression of granzyme B, by T, NKT-like CD28 ± subsets and NK cells were determined using flow cytometry.

**Results:**

There was a significant decrease in the percentage of T, NKT-like subsets and NK cells producing IFNγ, TNFα and granzyme B in all subjects in the presence of anti-CD137 blocking antibody compared with PHA alone (eg, 60% decrease in CD8 + granzyme B + cells) or MP. Stimulatory anti-CD137 was associated with an increase in the percentage of pro-inflammatory/cytotoxic cells. The inhibitory effect of anti-CD137 on IFNγ, TNFα and granzyme B production by CD28null cells was greater than by CD28+ cells.

**Conclusions:**

Blocking CD137 expression is associated with downregulation of IFNγ, TNFα and granzyme B by CD8+ T and NKT-like and NK cells. Targeting CD137 may have novel therapeutic implications for patients with COPD.

## Background

Chronic obstructive pulmonary disease (COPD) is a leading cause of death world wide. Existing treatments are largely symptomatic and the only approved anti-inflammatory medication, corticosteroids, has no proven disease modifying effect [1]. The mechanisms underlying this resistance are largely unknown, but suggest the presence of some self-maintaining pathogenic process, probably initiated by cigarette smoke, that prevents the normal resolution of the inflammatory response [2]. Our group has reported increased production of Th1 pro-inflammatory cytokines IFN-γ and TNF-α by CD8+ T cells in peripheral blood and lungs [3] and higher levels of the cytotoxic mediators granzyme b and perforin in peripheral blood in current and ex-smoker COPD patients compared to healthy smokers and never-smokers [4].

Our previous studies have focused on identifying the lymphocyte subset/s resistant to current therapeutics. We have shown that COPD is associated with increased CD8/CD28null cells in both current and ex-smoker COPD groups and these cells expressed more IFNγ, CD137 (4-1BB), granzyme B and perforin when stimulated than CD8 + CD28+ cells [5]. Using a mouse model of COPD, in mice exposed to cigarette smoke for 12 weeks, CD8/CD28null T-cells were significantly increased in the airway with a trend for an increase in lung tissue and blood [5].

NKT-like cells are a small but important subset of lymphocytes expressing both T cell and NK cell markers. Recently we also showed increased production of granzyme B and decreased inhibitory receptor CD94 by peripheral blood NKT-like and NK cells in COPD [6]. Furthermore NKT-like and NK cells were increased in bronchoalveolar lavage (BAL) of COPD patients and this was associated with increased NK cytotoxicity and decreased expression of the inhibitory receptor CD94 by both cell types [6]. As signaling through NK-cell associated CD137 has been shown to stimulate NK cell proliferation and IFNγ production [7], we hypothesized that we could target these pro-inflammatory/cyotoxic T, NKT-like and NK cells in COPD by inhibiting co-stimulation through CD137. Interestingly, we have recently shown that NKT-like cells lose CD28 expression and up-regulate CD137 following stimulation through the CD3 receptor in lung transplant patients diagnosed with bronchiolitis obliterans syndrome [8].

To investigate this hypothesis, we determined whether NKT-like and NK cells from COPD patients produce increased levels of pro-inflammatory cytokines and whether NKT-like cells up-regulate CD137 with concomitant loss of CD28. To determine the effect of blocking co-stimulation through CD137, isolated peripheral blood mononuclear cells (PBMC) from COPD patients and healthy controls were stimulated with phytohaemagglutinin (PHA) ± blocking anti-CD137 ± 10^-6^ M methylprednislone (MP) (±stimulatory anti-CD137 and isotyped matched control antibodies) and cytokine profiles and granzyme B expression by T, NKT-like and NK cells were determined using flow cytometry.

## Methods

### Patient and control groups

COPD volunteers were specifically recruited for the study and informed consent obtained. There was no exacerbation of COPD for 6 weeks prior. Subjects with other co-existing lung disease or aged greater than 75y were excluded. Ethics approval was obtained from the Royal Adelaide Hospital and the experiments were conducted with the understanding and the written consent of each participant. COPD was diagnosed using the GOLD criteria with clinical correlation (Mild COPD: FEV1/FVC < 70% but FEV1 ≥ 80% predicted; Moderate COPD FEV1 50% ≤ 80% predicted, Severe COPD FEV1 30% ≤ 50% predicted, very severe COPD FEV1 < 30%) [9]. Blood was collected from 10 patients with COPD (Table 1) of whom all were ex-smokers (at least one year). No patients were receiving oral GCS.

**Table 1:** Demographic details of the COPD and control group

**Subjects**	**Controls**	**COPD**
No. of subjects	14	10
Age (years)	44 (±8)	58 (±16)*
FEV1, % pred	110.4 (±9)	60.5 (±20)
FEV1, % FVC	96 (±12)	58 (±15)*
Male/Female	8/6	6/4

Data showing mean ± SD.

*Abbreviations*: *FEV1* forced expiratory volume in 1 second, *FVC* forced vital capacity, *P < 0.05 compared to controls.

Blood was also obtained from 14 non-smoking volunteers (Table 1) with normal lung function. These were healthy, recruited volunteers with no history of airways disease. All subjects underwent spirometry as part of their routine clinical assessment. Venous blood was collected into 10 U/mL preservative free sodium heparin (DBL, Sydney, Australia) and maintained at 4°C until processing. All patients were submitted to the same protocol and analysis performed retrospectively.

### T, NKT-like and NK cell percentages

T, NKT-like and NK cell percentages in blood from COPD patients and healthy controls were enumerated as previously reported [10].

### CD8 expression on NKT-like cells

We have previously shown an increase in CD8 + CD3+ T cells in blood from COPD patients compared with healthy controls. To enumerate CD8+ and CD8- subsets of NKT-like cells, 150 μL aliquots of heparinised blood were stained from COPD patients and healthy controls as previously reported [10].

### CD137 expression on NKT-like and NK cells

We have previously shown loss of CD28 (ie, an increase in the proportion of CD28null cells) and up-regulation of CD137 on CD28 null T cells from blood in patients with COPD compared with healthy controls [5].

CD137 is not constitutively expressed on T cells, but is up-regulated following initial T-cell activation [11]. To determine possible loss of CD28 and expression of CD137 on NKT-like and NK cells, 150 μL aliquots of blood were stimulated with phorbol myristate (25 ng/mL) (Sigma, Sydney, Australia) and ionomycin (1 μg/mL) (Sigma). Brefeldin A (10 μg/mL) was added as a “Golgi block” (Sigma) to prevent shedding of CD137 and the tubes incubated in a humidified 5% CO_2_/95% air atmosphere at 37°C [5]. Expression of CD137 was determination as previously reported for T cells [5]. Briefly, at 16 h 100 μL 20 mM EDTA/PBS was added to the culture tubes which were vortexed vigorously for 20 sec to remove adherent cells. Cells were permeabilized as described above. Two mL 0.5% bovine serum albumin (Sigma/Aldrich, Sydney, Australia)/Isoton II (Beckman Coulter, Sydney, Australia) was then added and the tubes centrifuged at 300 × g for 5 min. After decanting supernatant, Fc receptors were blocked with 10 μL human immunoglobulin (Intragam, CSL, Parkville, Australia) for 10 min at room temperature. Five μL of appropriately diluted anti- CD137 PE (BD), CD3 perCP.CY5.5 (BD), CD28 PE.CY7 (BD), CD56 APC (Beckman Coulter, Sydney, Australia), CD8 APC.CY7 (BD) and CD45 V500 (BD) monoclonal antibodies were added for 15 min in the dark at room temperature. Two mL of 0.5% bovine serum albumin (Sigma)/Isoton II (Beckman Coulter) was then added and the tubes centrifuged at 300 × g for 5 min. After decanting, cells were analyzed within 1 h on a FACSCanto II flow cytometer using FACSDiva software (BD). Samples were analyzed by gating lymphocytes using CD45 staining versus side scatter (SSC). A minimum of 350,000 low SSC events were acquired in list-mode format for analysis. NKT-like cells were identified as CD3 + CD56+ CD45+ low FSC/SSC events and NK cells as CD3-CD56+ CD45+ low FSC/SSC events.

### Granzyme B expression in CD28+ and CD28null NKT-like cells

PBMC were isolated from blood by standard density gradient centrifugation and cells re-suspended at 5 × 10^5^ mL in RPMI 1640 medium (Gibco, New York, USA) supplemented with 125 U/mL penicillin and 125 U/mL streptomycin (Gibco). To investigate NKT-like cell production of granzyme B, 150 uL of PBMC was added to FACS tubes. Cells were permeabilised by addition of 0.5 mL 1:10 diluted FACSperm (BD) to each tube, mixed, and incubated a further 10 min at room temperature in the dark. Two mL 0.5% bovine serum albumin (Sigma) in IsoFlow (Beckman Coulter) was then added and the tubes centrifuged at 300 × g for 5 min. After decanting supernatant, Fc receptors were blocked with 10 μL human immunoglobulin (Intragam, CSL, Parkville, Australia) for 10 min at room temperature. Five μL of appropriately diluted granzyme B FITC (BD), CD3 perCP.CY5.5 (BD), CD28 PE.CY7 (BD), CD56 APC (Beckman Coulter, Sydney, Australia), CD8 APC.CY7 (BD) and CD45 V500 (BD) monoclonal antibodies were added for 15 min in the dark at room temperature. Cells were analyzed within 1 h on a FACSCalibur flow cytometer using CellQuest software (BD).

### IFNγ and TNFα expression by NK cells and CD28+ and CD28null NKT-like cells

Production of IFNγ and TNFα by CD28+ and CD28null NKT-like cells was determined on blood samples from all subjects as previously reported [8].

### IFNγ and TNFα expression by CD137+ NK cells and CD137+ CD28+ and CD28null NKT-like cells

To determine if CD137 is expressed on NK and CD28+ and CD28null NKT-like cells that produce IFNγ and TNFα (and hence potential targets of blocking CD137), the percentage of these cells were determined from COPD patients and controls as previously described [8].

### Leucocyte stimulation with PHA ± antiCD137

To determine the effects of blocking co-stimulation through CD137 on T cells, NKT-like and NK cells, PBMC from COPD patients and control subjects were stimulated with 1 μg/mL PHA (Sigma, Sydney, Australia). For co-stimulation blockade, PBMC were activated with 1 μg/mL PHA alone or in the presence of 10 μg/mL purified blocking antibodies for CD137 (Biolegend, clone 4B4-1) and isotype-matched control antibodies (eBioscience, Sydney, Australia). Stimulation of T cells, NKT-like and NK cells through CD137 was performed with 10 μg/mL stimulatory antibodies for CD137 (R&D Systems, AF838).

### T, NKT-like and NK cell cytokine and granzyme B production following stimulation with PHA ± blocking anti-CD137

On the 4^th^ day post-activation with PHA ± blocking anti-CD137, cultures were stimulated with phorbol myristate (25 ng/mL) (Sigma, Sydney, Australia) and ionomycin (1 μg/mL) (Sigma). Brefeldin A (10 μg/mL) was added as a “Golgi block” (Sigma) and the tubes re-incubated in a humidified 5% CO_2_/95% air atmosphere at 37°C for 16 h. Cytokine and granzyme B determination was performed as previously reported [8]. Five μL of appropriately diluted anti- IFNγ FITC (BD), granzyme B V450 (BD), CD137 PE (BD), CD3 perCP.CY5.5 (BD), CD28 PE.CY7 (BD), CD56 APC (Beckman Coulter, Sydney, Australia), CD8 APC.CY7 (BD), TNFα V450 (BD) and CD45 V500 (BD) monoclonal antibodies were added for 15 min in the dark at room temperature. Two mL of 0.5% bovine serum albumin (Sigma)/Isoton II (Beckman Coulter) was then added and the tubes centrifuged at 300 × g for 5 min. After decanting, cells were analyzed as above.

### Effect of 10^-6^ M MP on T, NKT-like and NK cell cytokine and granzyme B production following stimulation with PHA ± blocking antiCD137

To determine the effects of 10^-6^ M MP on T and NKT-like cell cytokine and granzyme B production following stimulation, blood was stimulated with 1 μg/mL PHA (Sigma) ±10^-6^ M MP ± blocking antiCD137 for 4 days and processed as above.

### Statistical analysis

Statistical analysis was performed using the Mann–Whitney test. Correlations were performed using Spearman Rho correlation tests. SPSS software was applied and differences between groups of p < 0.05 considered significant.

## Results

### T, NKT-like and NK cell percentages

There were no significant changes in T, NKT-like or NK cells in blood from patients with COPD compared with control subjects (73 ± 12 and 76 ± 12% T cells (mean ± SD); 4.1 ± 4 and 3.5 ± 5% NKT-like cells; and 15.2 ± 5 and 10.5 ± 8% NK cells for COPD patients and control groups respectively). There was no change in absolute numbers of lymphocytes but there was an increase in the percentage and absolute numbers of CD8 T cells and a decrease in the percentage of CD4+ T cells in COPD patients compared with control group consistent with our previous reports [3-6,9,10] (data not shown).

### CD8+ and CD8-CD28null NKT-like cells

**Figure 1:**
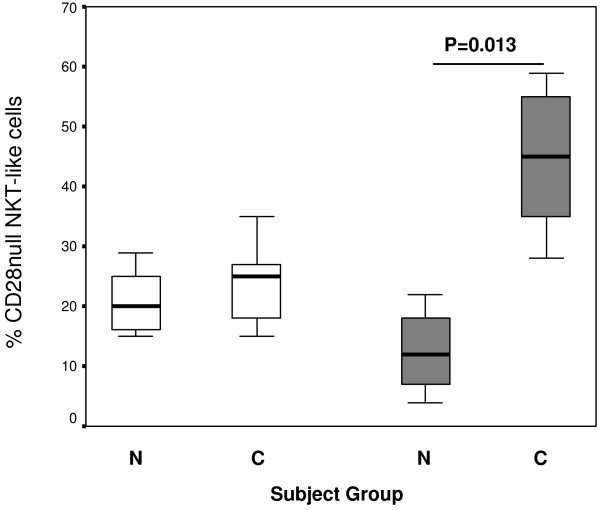
**CD28nullCD8- (clear bars) and CD28nullCD8+ NKT-like cells (grey bars) in 10 control subjects (N) and 14 patients with COPD (C).** Data presented as box plots. There was a significant increase in the percentage of CD28null/CD8+ NKT-like cells in patients with COPD compared with control subjects but no change in CD28null/CD8- NKT-like cells between groups.

There was a significant increase in the percentage of CD8+ NKT-like cells in patients with COPD compared with control subjects (59 ± 8 and 35 ± 6% (mean ± SD) for COPD patients and control groups respectively).There was a significant increase in the percentage of CD28null/CD8+ NKT-like cells in patients with COPD compared with control subjects (Figure 1) but no significant change for CD28null/CD8- NKT-like cells between groups (p > 0.05).

### Granzyme B expression by CD28+ and CD28null NKT-like cell subsets

**Figure 2:**
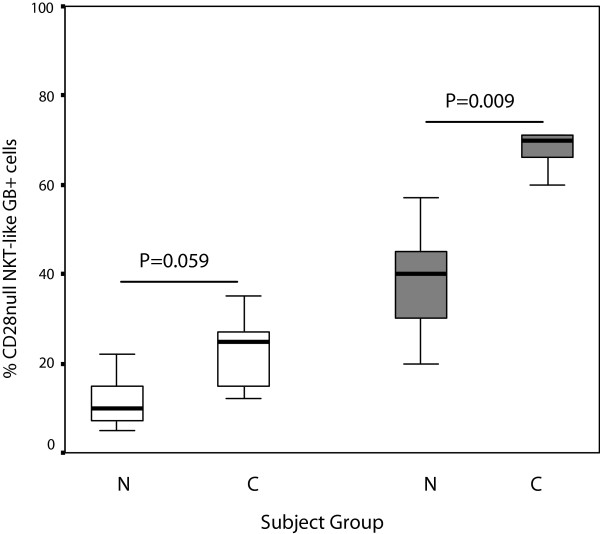
**CD28nullCD8- (clear bars) and CD28nullCD8+ NKT-like cells (grey bars) producing granzyme B (GB+) in 10 control subjects (N) and 14 patients with COPD (C).** Data presented as box plots. There was a significant increase in the percentage of CD28null/CD8 NKT-like cells and a trend for an increase in CD28null/CD8- NKT-like cells expressing granzyme B in patients with COPD compared with control subjects.

We have shown that COPD is associated with an increase in NKT-like cells producing granzyme B compared with control subjects [6]. Cells were subsequently stained to determine if CD28+ and/or CD28null NKT-like subsets are associated with increased granzyme B expression. There was a significant increase in the percentage of CD28null/CD8 + NKT-like cells (but not CD28null/CD8- cells) expressing granzyme B in patients with COPD compared with controls (Figure 2). There were no significant changes in the percentage of either CD28 + CD8- or CD28 + CD8+ subsets expressing granzyme B between either group (p > 0.05 for all) (data not shown).

### IFNγ and TNFα expression by CD28null and CD28+ NKT-like cell subsets

**Figure 3:**
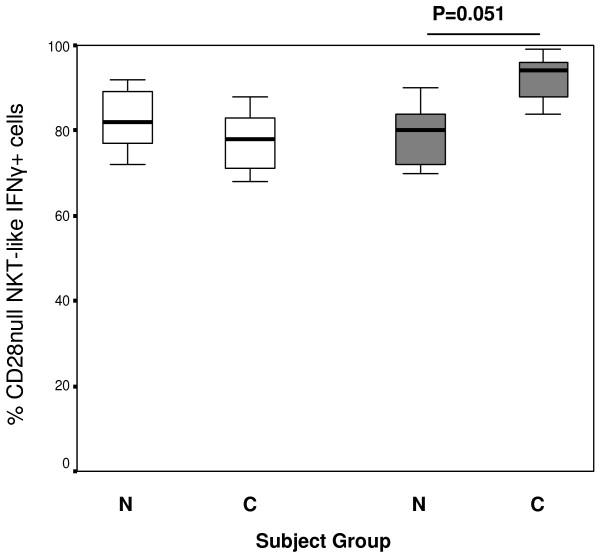
**CD28nullCD8- (clear bars) and CD28nullCD8+ NKT-like cells (grey bars) producing IFNγ in 10 control subjects (N) and 14 patients with COPD (C).** Data presented as box plots. There was a trend for an increase in the percentage of CD28null/CD8+ NKT-like cells producing IFNγ in patients with COPD compared with control subjects.

**Figure 4:**
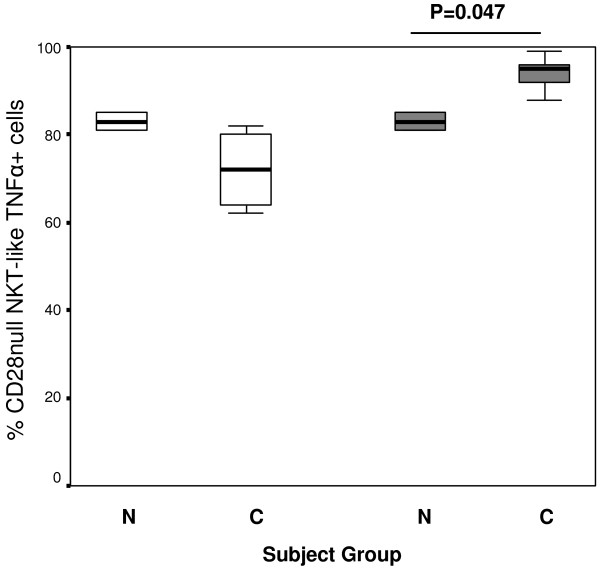
**CD28nullCD8- (clear bars) and CD28nullCD8+ NKT-like cells (grey bars) producing TNFα in 10 control subjects (N) and 14 patients with COPD (C).** Data presented as box plots. There was a significant increase in the percentage of CD28null/CD8+ NKT-like cells producing TNFα in patients with COPD compared with control subjects.

We have shown an increase in the percentage of CD8 + CD28null T cells producing IFNγ and TNFα in COPD patients compared with healthy controls [5]. In the current experiments, we determined if CD28+ and/or CD28nullNKT-like subsets are associated with increased IFNγ and TNFα production. CD28 expression was unaltered on either CD8- or CD8 NKT-like cell subsets following stimulation over this time period. There was however a trend for an increase in the production of IFNγ by CD28null/CD8+ NKT-like cells (but not CD28null/CD8- NKT-like cells) in patients with COPD compared with healthy controls (Figure 3).There was an increase in the percentage of CD28null/CD8+ NKT-like cells producing TNFα in patients with COPD compared with healthy controls (Figure 4). There were no significant changes in the percentage of CD28+/CD8- or CD28+/CD8+ producing IFNγ or TNFα between any of the groups studied (p > .05 for all) (data not shown).

### CD137 expression on NK cells and CD28null and CD28+ NKT-like cell subsets following stimulation

**Figure 5:**
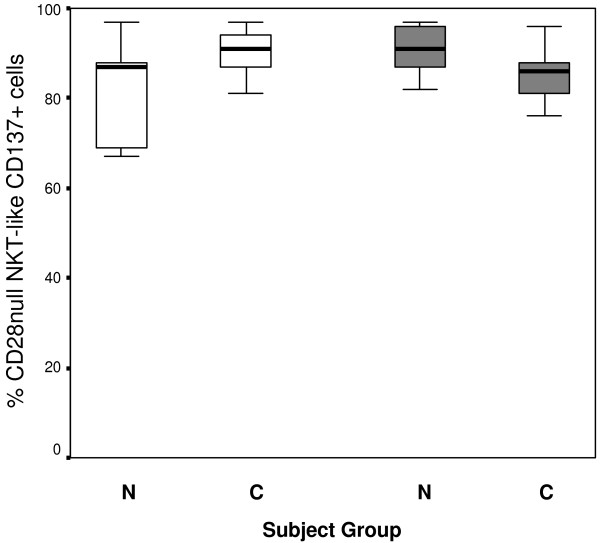
**CD28nullCD137 + CD8- (clear bars) and CD28nullCD137 + CD8+ NKT-like cells (grey bars) in 10 control subjects (N) and 14 patients with COPD (C).** Data presented as box plots. There were no significant differences in the percentages of CD28nullCD137 + NKT-like cell subsets between any of the groups (p > 0.05 for all).

**Figure 6:**
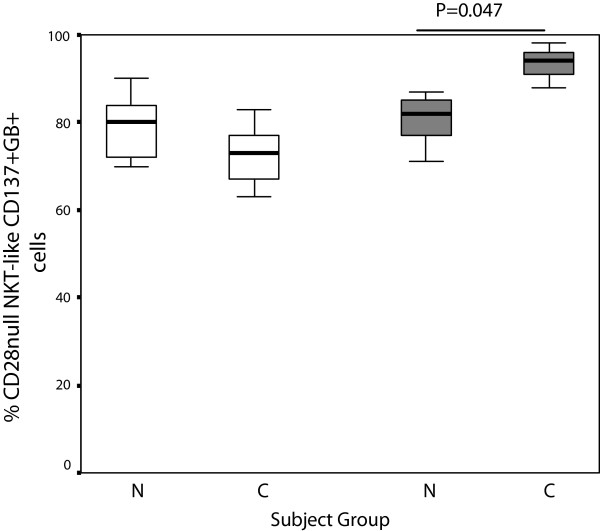
**CD28nullCD137 + CD8- (clear bars) and CD28nullCD137 + CD8+ NKT-like cells (grey bars) producing granzyme B in 10 control subjects (N) and 14 patients with COPD (C).** Data presented as box plots. There was a significant increase in CD28nullCD137 + CD8+ NKT-like cells producing granzyme B in patients with COPD compared with controls.

**Figure 7:**
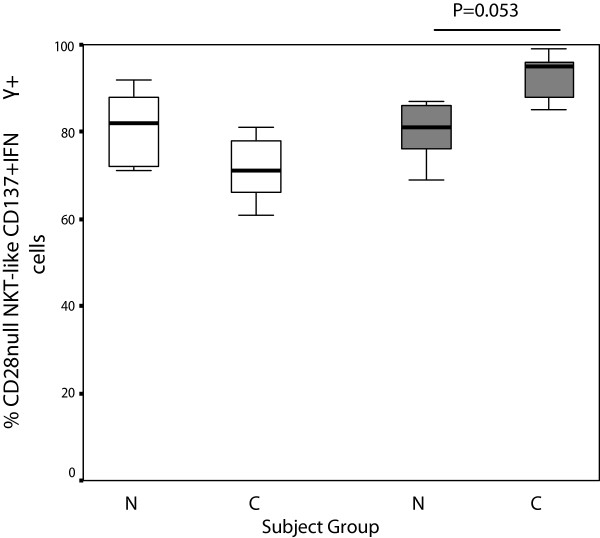
**CD28nullCD137 + CD8- (clear bars) and CD28nullCD137 + CD8+ NKT-like cells (grey bars) producing IFNγ in 10 control subjects (N) and 14 patients with COPD (C).** Data presented as box plots. There was a trend for an increase in CD28nullCD137 + CD8+ NKT-like cells producing IFNγ in patients with COPD compared with control subjects.

**Figure 8:**
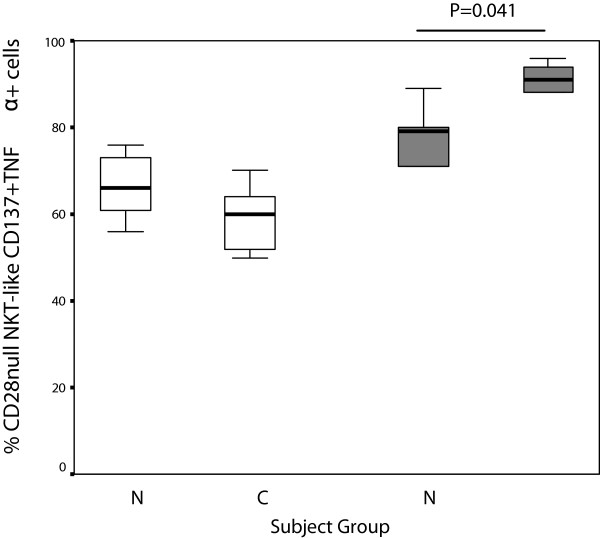
**CD28nullCD137 + CD8- (clear bars) and CD28nullCD137 + CD8+ NKT-like cells (grey bars) producing TNFα in 10 control subjects (N) and 14 patients with COPD (C).** Data presented as box plots. There was a trend for an increase in CD28nullCD137 + CD8+ NKT-like cells producing TNFα in patients with COPD compared with control subjects.

**Figure 9:**
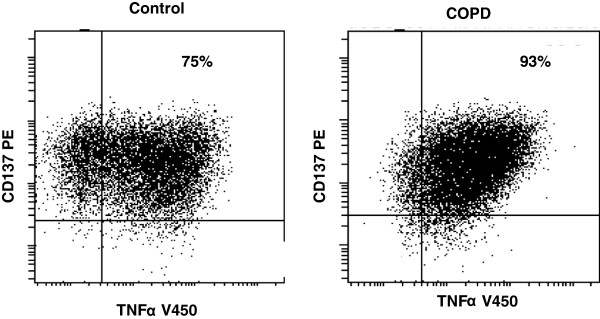
**Representative plots of CD28nullCD137 + CD8 + NKT-like cells producing TNFα from a control subject and a patient with COPD.** There was a significant increase in CD28nullCD137 + CD8+ NKT-like cells producing TNFα in patients with COPD compared with control subjects.

We have reported a loss of CD28 and up-regulation of CD137 on CD28null T cells in patients with COPD compared with healthy controls [5] findings consistent with the current study (results not shown). To investigate whether CD137 is expressed on NK cells and if there is a similar loss of CD28 and up-regulation of CD137 on NKT-like cells, blood was stimulated and processed as previously reported [5]. There was no difference in CD137 expression on CD28null or CD28+ CD8- or CD8+ NKT-like cells between the various groups studied (Figure 5.) There was however a significant increase in the co-expression of CD137 and granzyme B (Figure 6), a trend for an increase in CD137 and IFNγ (Figure 7) and a significant increase in CD137 and TNFα (Figure 8) in patients with COPD compared with controls. Representative plots of CD28nullCD8 + CD137 + NKT-like cells producing TNFα from a control subject and a patient with COPD are shown in Figure 9. There were no significant changes in the percentage of CD28+/CD8-/CD137+ or CD28+/CD8+/CD137+ NKT-like cells producing granzyme B, IFNγ or TNFα between either group studied (p > .05 for all) (data not shown). There was no significant change in the expression of CD137 on NK cells between COPD patients and control subjects (91 ± 7 and 87 ± 12% (mean ± sd) CD137 + NK cells for COPD patients and controls respectively). There was however a significant increase in CD137 + Granzyme b + expression in NK cells in COPD patients compared with control subjects (91 ± 8 and 62 ± 14% (mean ± sd) CD137 + GB + NK cells for COPD patients and controls respectively) but not CD137 + IFNγ + NK cells or CD137 + TNFα + NK cells (data not shown).

### Inhibitory effect of blocking anti-CD137 ± MP on granzyme b, IFNγ and TNFα expression by CD28null/CD28+ T cell subsets

Having shown a loss of CD28 and up-regulation of CD137 on CD28null T cells in patients with COPD compared with healthy controls [5] we then aimed to determine the effect of blocking antibody to CD137 on granzyme B expression and IFNγ and TNFα production by NK cells and CD28nullCD8- and CD28nullCD8+ T cells and NKT-like cells. The inhibitory effect of blocking anti-CD137 was significantly greater than for 10^-6^ M MP and CD28nullCD8+ and CD8- subsets than for CD28 + CD8+ and CD8- subsets for granzyme B, IFNγ and TNFα. Data for CD8+ T cells shown in Table 2. Data for CD8- T cells not shown. The inhibitory effect of blocking anti-CD137 and MP on granzyme B, IFNγ and TNFα expression was additive.

**Table 2:** **The inhibitory effect of anti-CD137 blocking antibody (10 μg/mL) (a137), 10**^**-6**^ **M methylprednisolone (MP) alone and in combination on granzyme B, IFNγ and TNFα expression by CD28null(28-) and CD28 + (28+) CD8+ T cells following stimulation (mean ± SEM of 8 experiments) compared with control antibody**

	**Granzyme B**		**IFNγ**		**TNFα**	
	**CD8+**		**CD8+**		**CD8+**	
	**28-**	**28+**	**28-**	**28+**	**28-**	**28+**
**a137**	**26** ± 4*	**17** ± 3	**28** ± 3*	**21** ± 2	31* ± 5	**23** ± 2
**MP**	**2** ± 1	**5** ± 2	**2** ± 1	**5** ± 2	**1** ± 1	**4** ± 2
**a137 + MP**	**29** ± 3^#^	**21** ± 3^#^	**31** ± 3^#^	**26** ± 3^#^	**32** ± 3^#^	**27** ± 3^#^

*The percentage inhibition of granzyme B, IFNγ and TNFα by CD28- and CD28+T cells compared with control (same specimen) without treatment/s.

The inhibitory effect of anti-CD137 was greater than for MP for granzyme B, IFNγ and TNFα in all T-cell subsets (p < 0.05 for all).

*The inhibitory effect of anti-CD137 was greater than for the CD28nullCD8+ subset than for the CD28 + CD8+ subset for granzyme B, IFNγ and TNFα (p < 0.05 for all).

^#^The combination of anti-CD137 and 10^-6^ M methylprednisolone resulted in the additive inhibition of granzyme B, IFNγ and TNFα expression in CD28null and CD28+ CD8+ T cells.

### Inhibitory effect of blocking anti-CD137 ± MP on granzyme b, IFNγ and TNFα expression by CD28null/CD28+ NKT-like cell subsets

The inhibitory effects of blocking anti-CD137 on granzyme B, IFNγ and TNFα expression by CD28null and CD28+ NKT-like cell subsets were similar to those for CD28null and CD28+ T cell subsets (Table 3). The inhibitory effect of blocking anti-CD137 was significantly greater than for CD28nullCD8+ and CD8- subsets than for CD28 + CD8+ and CD8- subsets for granzyme B, IFNγ and TNFα. Data for CD8+ NKT-like cells shown in Table 3. Data for CD8- NKT-like cells not shown. The inhibitory effect of blocking anti-CD137 and 10^-6^ M MP on granzyme b, IFNγ and TNFα expression was additive.

**Table 3:** **The inhibitory effect of anti-CD137 blocking antibody (10 μg/mL) (a137), 10**^**-6**^ **M methylprednisolone (MP) alone and in combination on granzyme B, IFNγ and TNFα expression by CD28null(28-) and CD28 + (28+) CD8+ NKT-like cells and NK cells following stimulation of PBMC from COPD patients (mean ± SEM, mean of 8 experiments) compared with control antibody**

	**Granzyme B**			**IFNγ**			**TNFα**		
	**CD8 + NKT-like**		**NK**	**CD8 + NKT-like**		**NK**	**CD8 + NKT-like**		**NK**
	**28-**	**28+**		**28-**	**28+**		**28-**	**28-**	
**a137**	**21** ± 2*	**14** ± 2	**14** ± 2	**26** ± 3*	**20** ± 3	**22** ± 3	30* ± 4	23* ± 4	**24** ± 3
**MP**	**1** ± 1	**3** ± 2	**2** ± 2	**2** ± 1	**5** ± 6	**43** ± 3^	**2** ± 1	**2** ± 1	**47** ± 9^
**a137 + MP**	**22** ± 4^#^	**17** ± 3^#^	**15** ± 3^#^	**28** ± 3^#^	**25** ± 2^#^	**66** ± 3	**32** ± 4^#^	**25** ± 4^#^	**70** ± 7^#^

*The percentage inhibition of granzyme B, IFNγ and TNFα by CD28- and CD28+ NKT-like and NK cells compared with control (same specimen) without treatment/s.

The inhibitory effect of anti-CD137 was greater than for MP for granzyme B, IFNγ and TNFα in all NKT-like cell subsets (p < 0.05 for all).

*The inhibitory effect of anti-CD137 was greater than for the CD28nullCD8+ NKT-like subset than for the CD28 + CD8+ NKT-like subset for granzyme B, IFNγ and TNFα (p < 0.05 for all). ^The inhibitory effect of MP on IFNγ and TNFα by NK cells was significantly greater than for Granzyme B (p < 0.05) and significantly greater than the effect of anti-CD137.

^#^The combination of anti-CD137 and 10^-6^ M methylprednisolone resulted in the additive inhibition of granzyme B, IFNγ and TNFα expression in CD28null and CD28+ CD8+ NKT-like cells and NK cells.

### Inhibitory effect of blocking anti-CD137 ± MP on granzyme b, IFNγ and TNFα expression by NK cells

The inhibitory effects of blocking anti-CD137 on granzyme b, IFNγ and TNFα expression by NK cells are shown in Table 3. The inhibitory effect of 10^-6^ M MP on IFNγ and TNFα by NK cells was significantly greater than for Granzyme B and significantly greater than the effect of MP on T and NKT-like IFNγ and TNFα expression.

### Effect of stimulatory anti-CD137 antibodies on granzyme b, IFNγ and TNFα expression by NK cells and CD28null/CD28+ T cells and NKT-like cell subsets

Granzyme b, IFNγ and TNFα production by NK cells and CD28null and CD28+ T cells and NKT-like cell subsets was significantly increased in the presence of stimulatory anti-CD137 antibodies compared with control antibody eg., 31 ± 7% and 22 ± 5% increase in TNFα by CD28nullCD8+ and CD28 + CD8+ T cells respectively.

## Discussion

In this study we show that COPD is associated with increased pro-inflammatory cytokine production by peripheral blood NK and NKT-like cells. We have previously shown that COPD is associated with increased granzyme B in peripheral blood NK and NKT-like cells and our current study extends these findings to show that these cells are both pro-inflammatory and cytotoxic [6]. Furthermore, down-regulation of CD28 on CD8+ NKT-like cells was associated with the most pro-inflammatroy/cytotoxic subset of these cells, a finding similar to that previously shown for CD28nullCD8+ T cells in patients with COPD [5]. Persistent antigenic stimulation has been shown to progressively and irreversibly down-regulate CD28 expression on CD8+ T cells [12]. Our new findings indicate that down-regulation of CD28 and up-regulation of CD137 is associated with increased pro-inflammatory cytokines and granzyme B production by CD8+ NKT-like cells. Furthermore, expression of CD137 on NK cells was associated with increased granzyme B by this lymphocyte subset. CD137 has previously been shown to be a stimulatory receptor for NK cells resulting in IFNγ production [7]. Signaling through NK cells-associated CD137 promoted helper function for CD8+ cytolytic T cells and may be involved in the crosstalk between innate and adaptive immunity [7]. Current therapeutics fail to inhibit these cytotoxic, pro-inflammatory molecules known to be associated with the disease process in COPD and our findings of a non-significant decrease in these molecules in CD8 + T and CD8 + NKT-like cells in the presence of MP confirm these clinical findings. In contrast, there was a significant decrease in IFNγ and TNFα by NK cells in the presence of MP compared with blocking anti-CD137 suggesting treatment with MP alone may be adequate at inhibiting these pro-inflammatory cytokines by NK cells. These results are consistent with a study showing a decrease in IFNγ production by isolated NK cells from COPD patients in the presence of budesonide [11]. The additive effect of MP with blocking anti-CD137 possibly reflects the different mechanisms of action of these two therapeutics, MP acting through the steroid receptor and anti-CD137 targeting the surface co-stimulatory molecule on these cells. Our findings of increased CD137 expression on NK cells and CD28null NKT-like cells and similar findings for CD28null T-cell subsets [5] allowed us to explore whether blocking this co-stimulatory receptor would be effective at inhibiting downstream effector mechanisms in all these pro-inflammatory/cytotoxic lymphocyte subgroups. Co-stimulatory signals through CD137 have been shown to preferentially induce CD8+ T cell proliferation and lead to the amplification of the cytotoxic T cell response [12] and may explain our previous and current findings for increased CD8+ cytotoxic cells compared with CD4+ T cell (and NKT-like cell) subsets. Pro-inflammatory cytokines, in particular TNFα may be the driving force behind COPD [13]. T-cell derived TNFα has been shown to cause apoptosis of airway epithelial cells and impair the clearance of these cells by alveolar macrophages [14]. We and others have shown that uncleared apoptotic material in the COPD airway can undergo secondary necrosis and thus perpetuate inflammation and this problem persists after smoking cessation [15]. TNFα has also been shown to induce IL-2Rs and IFNγ production by T cells and activate neutrophils, macrophages, endothelial cells and fibroblasts [16]; cells that play important roles in the pathogenesis of COPD [2]. Recently it has been shown that fractalkine, a potent chemoattractant for monocytes and T cells produced by airway smooth muscle cells, was induced in the presence of both IFNγ and TNFα [17].

These pro-inflammatory molecules may play a role in the systemic effects of the disease and associated co-morbidities such as cardiovascular disease, diabetes, osteoporosis and peptic ulceration [13]. Inhaled corticosteroids have major benefits for the treatment of airway inflammation in asthma but the reason for their relative lack of efficacy in COPD is both poorly understood and a major limiting factor in COPD treatment. Hence new effective treatments are urgently needed to help this devastating, widespread disease [2].

Blocking co-stimulation through CD137 resulted in a modest but significant reduction in these cytotoxic/pro-inflammatory molecules compared with MP alone, hence a more targeted approach with blocking anti-CD137 may prove more efficacious in treating patients with COPD where the use of corticosteroids has no proven disease modifying effect [2].

T cells have been shown to migrate to the lung and re-enter the circulation and as such, these cytotoxic cells identified in the peripheral blood of these patients may be reflective of cell populations in the lungs of these patients [18]. A study examining the presence of these pro-inflammatory cells T, NKT-like and NK cells in the airways and in intra-epithelial cells lining the lungs from these patients, as we have previously done for BAL [3] and bronchial brushings [19] would be an important addition to these studies. Furthermore, similar studies of current smokers with and without COPD may elucidate the effect of smoking on these pro-inflammatory/cytotoxic subsets.

In the mouse model, there have been several reports of blockade of the CD137 (4-1BB) pathway improving artherosclerosis [20] and autoimmune inflammation [21]. CD137-directed NK/NKT cells play an important role in the inflammatory response leading to the production of pro-inflammatory cytokines and cytolytic activity in septic shock [22] indicating efficacy and safety in the animal model. Furthermore, mice that were deficient in CD137 had substantially reduced levels of IFNγ and TNFα mRNA.

Interestingly, our findings show that these cytotoxic NK cells, and pro-inflammatory/cytotoxic CD28null T and NKT-like cells were present in healthy controls, albeit in reduced numbers to patients with COPD. These CD28null cells may also be responsible for other inflammatory conditions such as rheumatoid arthritis [23], cardiovascular disease [24], end-stage renal disease [25] and ulcerative colitis [26]. CD28null cells have also been reported in autoimmune disease where CD4 + CD28null cells were not susceptible to the regulatory effects of regulatory T cells [27], results similar to our own unpublished findings. This is an important point and may help explain the lack of regulatory control of these cells in all these inflammatory diseases. As such, these diseases may all benefit from our findings of therapeutic targeting of CD137. Furthermore monitoring response using these assays may offer a rapid indication of treatment efficacy.

## Conclusion

In conclusion, blocking CD137 expression in T, NKT-like and NK cells is associated with downregulation of IFNγ, TNFα and granzyme B and may have novel therapeutic implications for patients with COPD where current strategies are inadequate.

## Competing interests

The authors declare that they have no competing interests.

## Authors’ contributions

GH performed the concept and design of experiments, analysis and interpretation of data and manuscript preparation; MH supplied and characterized patient specimens and helped draft the manuscript; HJ supplied and characterized patient specimens and helped draft the manuscript; PNR supplied and characterized patient specimens and helped draft the manuscript; SH helped with study design, statistical analysis and helped draft the manuscript. All authors read and approved the final manuscript.

## Pre-publication history

The pre-publication history for this paper can be accessed here:

http://www.biomedcentral.com/1471-2466/14/85/prepub
